# Exoscope-assisted spine surgery: Insights from orthopedic and neurosurgical teams through a survey

**DOI:** 10.1016/j.bas.2026.105974

**Published:** 2026-02-13

**Authors:** Rossella Rispoli, Simona Legrenzi, Pietro Domenico Giorgi, Barbara Cappelletto

**Affiliations:** a Spine and Spinal Cord Surgery Unit, University Hospital of Udine, Italy; b Orthopedics and Traumatology Unit, Emergency and Urgency Department, A.S.S.T. Grande Ospedale Metropolitano Niguarda, Milan, Italy

**Keywords:** Exoscope-assisted spine surgery, Minimally invasive spine surgery, Neurosurgery, Orthopedic surgery

## Abstract

**Introduction:**

Spine surgery requires precise visualization to ensure safety in anatomically complex regions. Traditional microscopes, though effective, have ergonomic and communication limitations. High-definition three-dimensional (3D) exoscopes address these challenges by providing superior image quality, depth perception, ergonomics, collaboration, and teaching opportunities, while supporting minimally invasive techniques. This study reports the experience of two Italian centers adopting the exoscope as a primary visualization tool, integrating surgeons’ and staff perspectives through structured questionnaires.

**Methods:**

This retrospective, observational, dual-center study evaluated spinal procedures performed with the exoscope between January 2022 and June 2025 at two Italian institutions. Procedures included cervical, thoracic, and lumbar surgeries performed through anterior, posterior, and lateral approaches, and encompassed intradural and extradural pathologies. Clinical experience was complemented by two structured questionnaires, one for surgeons and one for non-surgeon team members, addressing image quality and visualization, ergonomics and fatigue, intraoperative communication and workflow, learning curve and implementation, and educational value.

**Results:**

A total of 880 spinal procedures were performed. Surgeons reported excellent visualization, illumination, magnification, depth perception, and ergonomics, with reduced fatigue and improved workflow. Competency was typically achieved within three cases. Non-surgeons confirmed improved image quality, intraoperative communication, awareness of surgical actions, and educational engagement. Both groups consistently endorsed the exoscope as a transformative surgical and teaching tool.

**Conclusions:**

The exoscope enhances visualization and ergonomics while fostering collaboration and education in the operating room. It offers significant advantages over traditional microscopes, improving precision, efficiency, and training across diverse spinal procedures, and represents a valuable tool for routine clinical practice.

## Highlights

• Exoscope provides superior visualization, depth perception, and illumination.
• Surgeons reported improved ergonomics with reduced fatigue and faster workflow.
• Competency in exoscope use was typically achieved within three surgical cases.
• Non-surgeon staff noted better communication, awareness, and engagement.
• Exoscope enhances surgical precision, efficiency, and educational value.

## 1 Introduction

Spinal surgery demands exceptional precision due to the complex anatomy and proximity to vital neural and vascular structures. Optimal visualization is essential to minimize complications and improve patient outcomes. Traditional operating microscopes, while effective, have limitations such as ergonomic constraints and limited shared visualization for the surgical team. The introduction of high-definition, three-dimensional exoscopic systems has addressed many of these challenges by providing superior image quality, enhanced depth perception, and a heads-up ergonomic design that reduces surgeon fatigue (Ricciardi et al., 2019; Moisi et al., 2019). Exoscopes also enable improved team collaboration and educational opportunities by projecting the surgical field on large monitors visible to all operating room members. Their versatility allows their use across a wide range of spinal procedures at any level, including anterior, lateral, and posterior approaches, facilitating safe dissection and precise decompression and stabilization. The enhanced visualization supports minimally invasive techniques, reducing tissue trauma, blood loss, and recovery times (Motov et al., 2021; Siller et al., 2020a).

This narrative review presents the combined experience of two Italian centers, composed of orthopedic surgeons and neurosurgeons, who have adopted exoscope technology as a primary visualization device in a wide range of spinal procedures. To complement the descriptive analysis, the perspectives of surgeons and surgical team members were collected through a structured questionnaire. The review evaluates the practical advantages, limitations, and overall feasibility of routine exoscope use across two distinct surgical specialties.

## 2 Materials and methods

This retrospective, observational, dual-center study evaluates the application of exoscopic visualization in spine surgery across two spine surgery centers.

### 2.1 Participating centers

Spine and Spinal Cord Surgery Unit, University Hospital of Udine, Italy and Orthopedics and Traumatology Unit, ASST Niguarda Metropolitan Hospital, Milan, Italy.

### 2.2 Device

A 3D Aesculap Aeos® robotic exoscope was used. Stereoscopic visualization required a dedicated display and polarized 3D glasses. Video recording and still image capture required dedicated 3D recording software, integrated into the central unit.

The operating room was equipped with two monitors: a 55-inch 3D 4K monitor and a 26-inch 3D monitor, both with High Dynamic Range (HDR). The screens were positioned facing each other so that two surgeons working opposite one another during spine surgery could maintain optimal viewing, while remaining clearly visible and accessible to all operating room personnel (including the anesthesiologist, scrub nurses, and technicians).

Conventional operating microscopes available at our institution were Leica F40 and Leica ARveo 8 (Leica Microsystems, Wetzlar, Germany).

### 2.3 Patient population

All spinal surgeries performed with the exoscope between January 2022 and June 2025 were included. The exoscope was used in 3D mode as the standard visualization setting in all cases. Procedures encompassed a broad spectrum of interventions, ranging from single-level decompressions to complex multilevel instrumentation, and involved both intradural and extradural pathologies. Spinal procedures were extracted and categorized by anatomical region (cervical, thoracic, and lumbar spine) and approach.

### 2.4 Data collection

Clinical and intraoperative data were retrospectively obtained from institutional surgical registries and operative reports.

### 2.5 Questionnaire

Questionnaires were administered between July and August 2025 and were not linked to individual surgical procedures; rather, responses reflect the overall experience of operating room personnel during exoscope-assisted surgeries.

A surgeon (S) questionnaire was administered to surgeons, assistant surgeons, and residents. It consisted of nineteen 5-point Likert scale items divided into five sections, along with one open-ended question for additional suggestions. The five sections addressed: image quality and visualization; ergonomics and fatigue; intraoperative communication and workflow; learning curve and implementation; and overall educational and didactic value.

A non-surgeon (NS) questionnaire was administered to scrub and circulating nurses, anesthesiologists, neurophysiologists, neurophysiology technicians, and radiology technicians. It consisted of seven 5-point Likert scale items divided into four sections, along with one open-ended question for additional suggestions. The four sections addressed: image quality and visualization; ergonomics and fatigue; intraoperative communication and workflow; and overall educational and didactic value.

## 3 Results

A total of 880 spinal procedures were performed across both centers using exoscopic visualization, including 245 cervical, 111 thoracic, and 524 lumbar surgeries. Of the cervical procedures, 128 were performed via an anterior approach, 70 via a posterior approach, and 47 via a combined (double) approach. Of the thoracic procedures, 101 were performed via a posterior approach and 10 via a lateral approach. Of the lumbar procedures, 438 were performed via a posterior approach and 86 via an antero-lateral approach.

The S questionnaire was completed by 13 surgeons or assistant surgeons, and 2 residents. The NS questionnaire was completed by 34 surgical team components: 18 scrub or circulating nurses, 3 anesthesiologists, 4 neurophysiologists, 1 neurophysiology technicians, and 8 radiology technicians.

### 3.1 S questionnaire results

Surgeons consistently reported high satisfaction with the exoscope's image quality and visualization. Resolution was rated as excellent by 73.3% and as very good by 26.7% of respondents. Magnification was judged excellent by 80% and very good by 20%, while illumination was considered excellent by 73.3%, very good by 20%, and good by 6.7%. Depth perception of the surgical field and visualization of deep anatomical structures were rated excellent by 73.3% and very good by 26.7%.

Ergonomic advantages were particularly appreciated during prolonged and complex procedures. Compared with the operating microscope, the exoscope reduced surgeon fatigue in 93.3% of cases (markedly in 80% and slightly in 13.3%) and improved intraoperative comfort in 93.4% (markedly in 86.7% and slightly in 6.7%). Tolerance of 3D glasses was reported as very good by 73.3% of respondents and good by 26.7%. No intraoperative conversions to a conventional operating microscope were required by 80% of surgeons, while 20% reported only very occasional conversion.

**Fig. 1:**
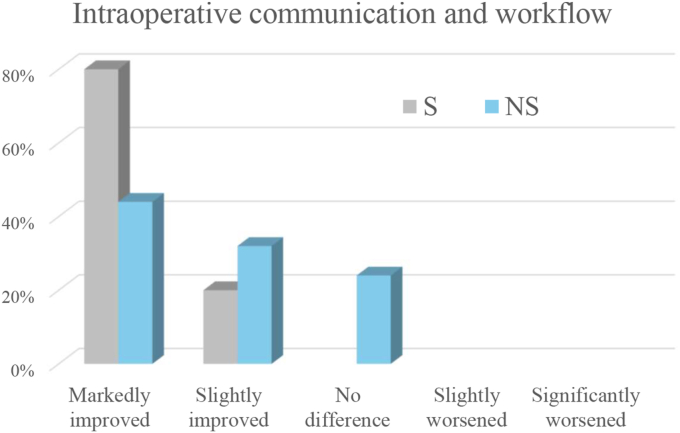
Ratings of intraoperative communication and workflow when using the exoscope compared with the operating microscope. The x-axis shows the improvement grade, and the y-axis shows the percentage of respondents selecting each grade. Bars are color-coded for surgeons (S) and non-surgeons (NS).

**Fig. 2:**
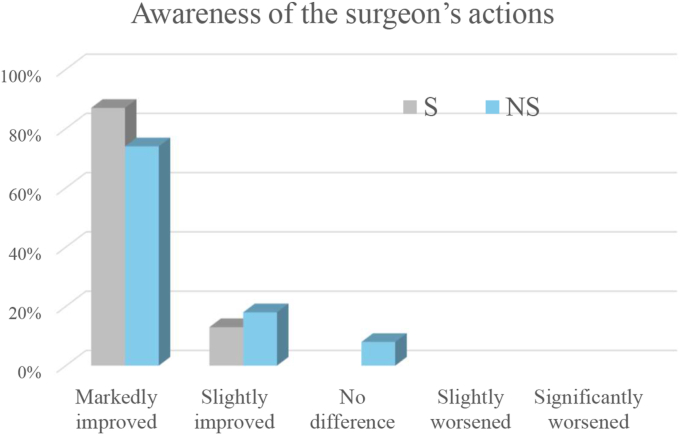
Ratings of awareness of the surgeon's actions when using the exoscope compared with the operating microscope. The x-axis shows the improvement grade, and the y-axis shows the percentage of respondents selecting each grade. Bars are color-coded for surgeons (S) and non-surgeons (NS).

Compared with the operating microscope, the exoscope was judged to improve intraoperative communication among surgical team members and workflow by all respondents (markedly in 80% and slightly in 20%), and to enhance awareness of the primary surgeon's actions by all assistant surgeons (markedly in 80% and slightly in 20%). The results are shown in Fig. 1, Fig. 2.

Surgeons reported that learning to operate with the exoscope was straightforward and the level of difficulty in learning was very easy (60%) or easy (26.7%). The learning curve for experienced surgeons was brief, with competency typically achieved within 3 cases (73.3%), 6 cases (20%), or 10 cases (6.7%). The use of the multifunctional robotic arm was rated as very easy (46.7%) or easy (53.3%). The integrated controls operated by hand or foot pedal, were considered very easy (20%) or easy (60%) or neutral (20%). The function that overlays previous images onto the current view was found to be very useful (46.7%), useful (33.3%), neutral (13.3%), or slightly useful (6.7%). The automatic camera repositioning feature was also rated very useful (73.3%), useful (20%), or slightly useful (6.7%). The system for processing and storing videos and images for clinical and educational purposes was found to be very useful (60%), useful (26.7%), or neutral (13.3%).

**Fig. 3:**
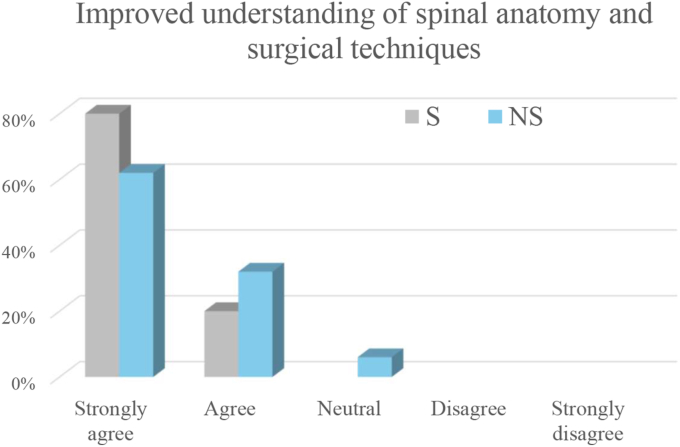
Ratings of agreement regarding improved understanding of spinal anatomy and surgical techniques when using the exoscope compared with the operating microscope. The x-axis shows the agreement grade, and the y-axis shows the percentage of respondents selecting each grade. Bars are color-coded for surgeons (S) and non-surgeons (NS).

**Fig. 4:**
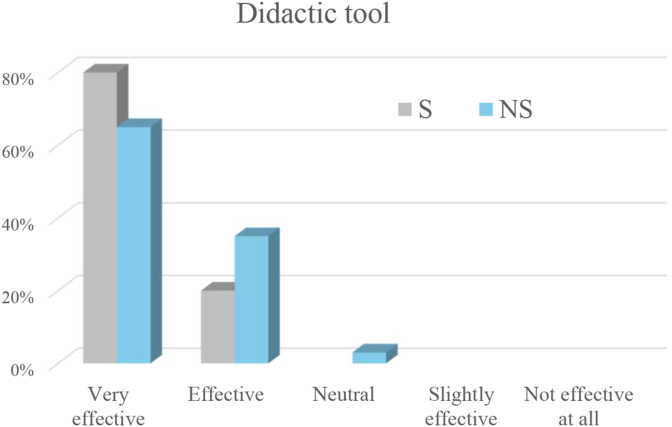
Ratings of the exoscope's efficacy as a didactic tool. The x-axis indicates efficacy grade, and the y-axis shows the percentage of respondents selecting each grade. Bars are color-coded for surgeons (S) and non-surgeons (NS).

The surgeons strongly agreed (80%) or agreed (20%) that the exoscope provides a better understanding of spinal anatomy and surgical techniques compared to the microscope (Fig. 3). They also considered the exoscope very effective (80%) or effective (20%) as a didactic tool (Fig. 4).

Among the comments provided in the open-ended question, we highlight the following: *“It made me dependent on the technology, and returning to the microscope was difficult.” “The advantages of using the exoscope are so significant that in our center all spinal decompression procedures are now exoscope-assisted, and a return to traditional visualization systems would be unthinkable and problematic.” “An indispensable tool for surgical advancement.”* All comments are available in the supplementary material.

### 3.2 NS questionnaire results

Non-surgeons reported excellent (41,2%), very good (44.1%), good (5.9%), fair (5.9%), or poor (2.9%) appreciation for the exoscope's image quality.

Tolerance of 3D glasses was very good (29.4%), good (29.4%), indifferent (32.4%), or poor (8.8%).

Compared with the operating microscope, the exoscope was judged to improve intraoperative communication and workflow among surgical team members markedly in 44.1% of cases, and slightly in 32.4%, while 23.5% reported no difference. It was also considered to enhance awareness of the surgeon's actions markedly in 73.5% of cases, and slightly in 17.6 with 8.8% reporting no difference. The results are shown in Fig. 1, Fig. 2.

The non-surgeons strongly agreed (61.8%) or agreed (32.4%) that the exoscope provides a better understanding of spinal anatomy and surgical techniques compared to the microscope, while 5.9% were neutral (Fig. 3). They also considered the exoscope very effective (64.7%) or effective (32.4%) as a didactic tool, with 2.9% neutral (Fig. 4). The exoscope was considered to greatly improve (44.1%), improve (32.4%), or have no effect (20.6) on active engagement in the procedure.

Among the comments provided in the open-ended question, we highlight the following: *“It would be useful to specify in the list whether the exoscope was employed to optimize the positioning of radiological equipment.” “I suggest providing the exoscope with a video output channel suitable for connection to intraoperative neurophysiology devices, so that the neurophysiologist can follow the procedure on their own screen.”* All comments are available in the supplementary material.

## 4 Discussion

This study reports the results from two Italian centers where spine surgery is performed by neurosurgical and orthopedic teams, showing that the implementation of exoscope technology was successful for both specialties. With 880 procedures performed using the exoscope, our data provide strong support for its feasibility, reproducibility, and broad clinical utility across cervical, thoracic, and lumbar procedures.

### 4.1 Image quality and visualization

One of the most significant findings from our experience is the high level of surgeon satisfaction with the exoscope's optical performance. Non-surgeons, through the questionnaire, also reported excellent to good appreciation for the exoscope's image quality. Across all anatomical regions, the exoscope consistently provides excellent image quality, depth of field, and magnification, resulting in improved intraoperative precision, particularly in anatomically complex or deep surgical fields (Motov et al., 2021). These findings are supported by the structured questionnaire responses, which indicated that surgeons highly valued the exoscope's image quality and overall visualization. Resolution was consistently rated as excellent or optimal, and magnification received similarly high ratings, reflecting the device's ability to provide detailed views of the surgical field. Illumination was also considered excellent or optimal, contributing to clear visualization throughout the procedure. Depth perception of the surgical field and the ability to visualize deep anatomical structures were likewise rated highly, underscoring the exoscope's effectiveness in enhancing clarity and stereoscopic perception, attributes that are particularly critical when operating in confined or anatomically complex regions. These features were regarded as significantly enhancing the visualization of complex anatomical structures across all spinal regions and surgical approaches (Moisi et al., 2019; Motov et al., 2021; Siller et al., 2020a).

### 4.2 Anatomical advantages, learning curve and implementation

In cervical spine surgery (n = 245), the exoscope proved especially advantageous in both anterior and posterior approaches. In anterior cervical discectomy and fusion (ACDF) or corpectomy, it facilitated safe dissection within narrow corridors bordered by delicate structures such as the esophagus and carotid sheath. Enhanced digital magnification enabled meticulous bony work, particularly in cases with ossified posterior longitudinal ligament (OPLL) or bulky osteophytes, where surgical precision is essential to avoid neural injury (Lin et al., 2021; Encarnacion Ramirez et al., 2021). Posterior cervical procedures also benefited significantly: the exoscope provided a panoramic, well-lit view of the interlaminar window and lateral recess, reducing the need for frequent repositioning commonly required with traditional operating microscopes (Lee et al., 2021). Its flexible angulation and ergonomic display allowed surgeons to maintain optimal posture throughout multilevel decompressions, procedures that are otherwise physically demanding and time-intensive.

In thoracic spine surgery (n = 111), the exoscope also proved highly useful. The lateral approach, long considered technically challenging due to field depth and the proximity of major vascular and visceral structures, was markedly enhanced by exoscopic visualization (Tan et al., 2025). In cases of burst fractures with retropulsed fragments or posterior thoracic disc herniations, the exoscope enabled stable, high-resolution visualization without the need for aggressive retraction or visceral mobilization. The broad depth of field and digital flexibility were particularly beneficial in minimally invasive thoracic procedures, facilitating safe decompression and tumor resection within narrow working corridors (Giorgi et al., 2020).

**Fig. 5:**
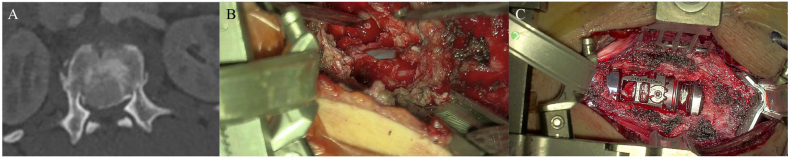
(A) Axial CT scan showing an L1 vertebral body fracture with a large endocanalar fragment. (B) Exoscopic intraoperative view demonstrating complete dural decompression (patient in right lateral decubitus, surgical approach from the left). The exoscope provided magnified, high-definition visualization with improved depth perception, facilitating safe decompression. (C) Exoscopic view of the cage in final position. The patient was then positioned supine for percutaneous fixation.

In lumbar surgery(n = 438), the most frequently performed spinal intervention, the exoscope's contributions were equally substantial. For decompression procedures, particularly bilateral decompression via unilateral approach, the angled optics allowed for precise contralateral foraminotomy with minimal neural retraction. In spinal fusion procedures (e.g., TLIF, PLIF), the exoscope supported accurate endplate preparation and graft placement while maintaining a stable visual field, even when paired with intraoperative fluoroscopy (D'Ercole et al., 2021; Peng et al., 2021). In the lateral position, it provided magnified, high-definition visualization with improved depth perception, thereby facilitating safe decompression. Fig. 5 shows an exoscopic intraoperative view demonstrating complete dural decompression (Ramirez et al., 2021).

**Fig. 6:**
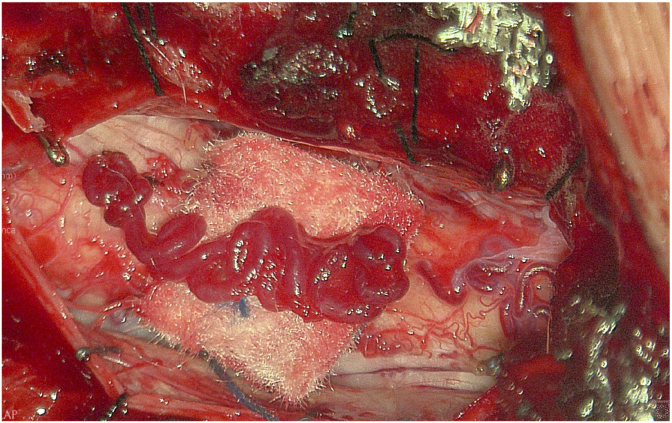
Exoscopic intraoperative view of a spinal dural arteriovenous fistula. The feeding vessel arises from the left L2 segmental radiculomeningeal artery with venous outflow toward T12. The exoscope provided high-definition magnification of dilated perimedullary veins along the conus medullaris and nerve roots. Fluorescein, combined with the integrated fluorescence filter, enhanced visualization of the vascular anatomy.

The exoscope also offers significant advantages in the management of intradural tumors, where its high-definition magnification and illumination enhance the visualization of neural and vascular structures, facilitating safe tumor dissection and minimizing neural manipulation (Calvanese et al., 2024; Carl et al., 2019). Fluorescence integration further improves intraoperative assessment of vascular anatomy and tumor boundaries (De la Garza-Ramos et al., 2014; Tonchev et al., 2025). Fig. 6 illustrates an exoscopic intraoperative view of a spinal dural arteriovenous fistula.

Surgeons found the exoscope easy to learn, with rapid attainment of proficiency and a brief learning curve: experienced surgeons typically achieved competency after only a few cases (Layard Horsfall et al., 2022a). A minority of surgeons (20%) reported occasional switching to a conventional operating microscope, predominantly during the early phase of exoscope adoption. This was not related to visualization failure or safety concerns, but rather to surgeon preference in selected procedural steps, particularly in highly delicate microvascular phases or due to long-standing familiarity with microscope-based techniques.

The robotic arm was consistently appreciated for its intuitive handling, seamless repositioning, and precise micromovements, which facilitated safe work in deep and narrow fields (Schupper et al., 2021a). Integrated hand and foot controls were likewise considered practical and user-friendly, allowing autonomous management of the surgical field. Advanced functions such as image overlay and automatic camera repositioning, particularly useful when the exoscope has to be moved away from the target, such as during intraoperative fluoroscopy, were regarded as highly useful for improving accuracy during tumor removal, vessel identification, and intraoperative fluoroscopy. The integrated system for video and image processing was also valued as a practical tool for both clinical documentation and teaching.

### 4.3 Ergonomics and fatigue

Beyond anatomical considerations, one of the most consistent themes emerging from both clinical use and questionnaire feedback was the improvement in surgical ergonomics. Ergonomic advantages were particularly appreciated during prolonged and complex procedures. The heads-up operating position afforded by the exoscope allowed surgeons to maintain a neutral spine posture, significantly reducing neck and shoulder strain. These ergonomic benefits are not merely anecdotal but have direct implications for sustained concentration, procedural efficiency, and long-term surgeon health, particularly relevant in complex or lengthy spine surgeries (Kwan et al., 2019; Oertel and Burkhardt, 2017).

### 4.4 Communication and workflow

**Fig. 7:**
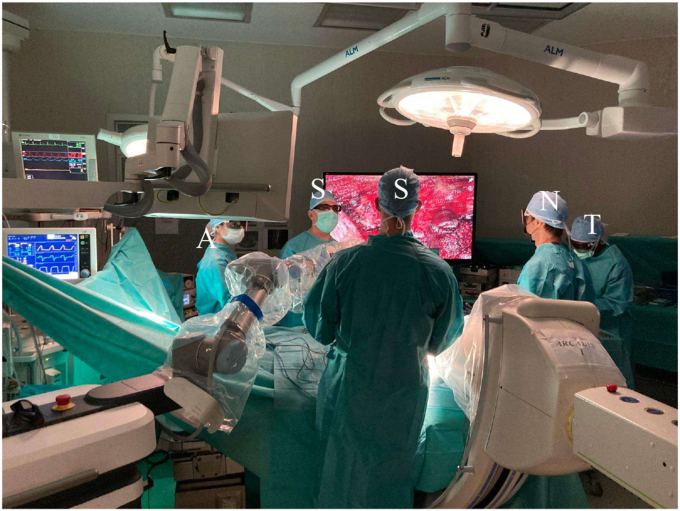
Operating room setup with the exoscope. Two surgeons (S) face each other, viewing the large monitors with their heads in a physiological posture. The anesthesiologist (A), scrub nurse (N), and X-ray technician (T), all wearing 3D glasses, actively observe the monitors, highlighting the coordinated participation of the entire surgical team throughout the procedure.

Both surgeons and non-surgeons consistently perceived the exoscope as a powerful tool for enhancing intraoperative communication and workflow. From the surgeons' perspective, the shared visualization significantly facilitated collaboration among team members and improved the assistants' awareness of the primary surgeon's maneuvers, leading to greater synchronization during critical steps of the procedure. Similarly, non-surgeons, including anesthesiologists, nurses, neurophysiologists, neurophysiology technicians, and radiology technicians, highlighted the educational and organizational benefits of the system. The real-time, three-dimensional shared visualization not only deepened their understanding of spinal anatomy and surgical techniques but also allowed them to engage more actively in the procedure. Unlike traditional microscopes, where the operative field is restricted to the primary surgeon, the exoscope projects high-definition images onto large monitors visible to the entire team. This feature was widely acknowledged as transformative, fostering a more collaborative and participatory operating room environment (Fig. 7). This collective access enhanced team coordination, situational awareness, and intraoperative communication, factors that contribute to smoother procedures and potentially better outcomes (Pott et al., 2025).

### 4.5 Educational and training impact

The educational value of the exoscope emerged as a key benefit in our study. Tutors regarded it as an optimal tool for guiding residents and fellows, as the shared visualization created a common reference point for instruction. Trainees consistently reported that the shared 3D view accelerated their understanding of spinal anatomy and procedural steps, effectively transforming the operating room into a dynamic teaching environment. Senior surgeons adapted quickly to the new system and emphasized the advantage of real-time teaching through a unified visual field. These findings align with recent literature underscoring the exoscope's potential in surgical education, where immersive visualization enhances learning and fosters more meaningful intraoperative teaching (Calloni et al., 2022; Layard Horsfall et al., 2022b).

### 4.6 Challenges of exoscope-assisted spine surgery

Despite the numerous advantages of exoscope-assisted spine surgery, including enhanced ergonomics, improved visualization, and educational benefits, several limitations must be addressed for its widespread adoption (Krishnan et al., 2017). One major concern is depth perception: although modern exoscopes offer 3D imaging, some surgeons report difficulty accurately interpreting spatial relationships, especially in deep or narrow anatomical corridors (Schupper et al., 2021b). While stereoscopic viewing with 3D glasses is a key feature of many exoscope systems, reliance on glasses may lead to discomfort, visual fatigue, and reduced concentration during lengthy procedures (Montemurro et al., 2022; Vattipally et al., 2024). At the same time, full-time stereoscopic visualization provides continuous depth perception across magnification changes and may allow surgeons to focus on the surgical task without repeatedly recalibrating depth cues during zoom adjustments or mentally compensating for 2D-to-3D transitions. Nonetheless, tolerance to stereoscopic visualization may vary among individuals and could be influenced by age and exposure duration. In this context, Heo et al. (2022) reported dizziness and asthenopia during prolonged 3D endoscopic surgery and suggested that intermittent 3D viewing or switching to 2D for non-critical steps may mitigate symptoms. Our study did not stratify tolerance by age or correlate symptoms with operative time. Newer exoscope platforms offering glasses-free 3D visualization may help reduce eye strain and visual fatigue. Another challenge is the potential overdependence on advanced technology, which might reduce opportunities for younger surgeons to develop traditional microsurgical skills (Langer et al., 2020). Economic barriers, including high initial costs and the need for specialized training, can further hinder adoption, particularly in resource-limited settings. Despite a relatively short learning curve, institutions must still invest time and resources in training surgical teams. Looking ahead, the integration of robotics and artificial intelligence may enhance exoscope performance by enabling real-time feedback, improved precision, and automated camera control (Muhammad et al., 2019). Advances in stereoscopic rendering, spatial depth cues, and augmented reality overlays could further improve intraoperative navigation and safety. However, large-scale, prospective studies comparing exoscopic and traditional approaches are essential to validate clinical outcomes, cost-effectiveness, and long-term benefits. With continued innovation and rigorous evaluation, the exoscope is poised to play an increasingly central role in spine surgery (Siller et al., 2020b; Schupper et al., 2021c).

### 4.7 Microscope versus exoscope

A key limitation of the current literature is the lack of robust, quantitative data directly comparing operating microscopes and exoscopes, a gap that is increasingly relevant as exoscopic systems are progressively integrated into spinal surgery practice. Exoscopes offer multiple advantages, including enhanced ergonomics, greater depth of field, team-wide visualization, and improved educational opportunities for trainees. Nevertheless, high-quality comparative evidence remains limited and heterogeneous, with most studies consisting of retrospective series, feasibility reports, or subjective surgeon assessments.

Recently, Du Preez et al. (Du Preez et al., 2025) conducted a qualitative assessment of exoscope-assisted surgery, offering valuable insights into visualization quality, ergonomics, workflow integration, and surgeon comfort in comparison with the operative microscope. However, their analysis primarily reflects subjective perceptions rather than objective, outcome-based measures. Vattipally et al. (2024) synthesized 31 studies (481 patients) and reported that, compared with the operating microscope, exoscope-assisted surgery was most consistently associated with improved surgeon posture/ergonomics, better trainee education, a more compact setup, and greater assistant participation, whereas findings regarding stereopsis, illumination, and cost were mixed across studies. Nawabi et al. (2024) reported that exoscope-assisted spine surgeries demonstrated reduced blood loss, shorter operative times, and decreased hospital stays compared with matched microscope cases. Other centers observed no significant differences in operative metrics, yet surgeons consistently reported improved comfort and ergonomic posture, even when visualization quality was perceived as inferior for certain procedures (Siller et al., 2020b).

Exoscope technology has rapidly evolved in recent years, and the available clinical evidence suggests that surgical outcomes and complication rates are generally comparable to those achieved with the conventional operating microscope. In a systematic review assessing surgical complications during exoscope-assisted procedures, 40 studies including 1328 patients were analyzed, reporting an overall complication rate of 2.6%; importantly, the complication profile was broadly similar to that reported for comparable procedures performed with the operating microscope (Montemurro et al., 2022). Overall, current evidence suggests the exoscope mainly improves surgical workflow (ergonomics and visualization) rather than outcomes, and further comparative studies are needed to determine whether these advantages translate into clinical benefit.

Contemporary operating microscopes allow projection of the surgical field onto large monitors, enabling the entire OR team to observe the procedure alongside the traditional ocular view. Exoscopes provide similar team-wide visualization, with additional benefits including ergonomic freedom, enhanced team participation, and improved educational opportunities. Hybrid platforms, such as the Zeiss Kinevo 900 and Pentero 800 S, allow seamless switching from digital exoscope mode to optical microscope mode, providing flexibility, workflow optimization, and potential cost-effectiveness in multi-use operating rooms. This field is evolving rapidly, with frequent technological updates improving visualization quality, ergonomics, and overall team engagement.

### 4.8 Future direction in exoscope-assisted spine surgery

Despite current limitations, the future of exoscope-assisted spinal surgery appears highly promising, with ongoing technological advancements poised to expand its clinical utility, enhance surgeon experience, and refine procedural efficacy (Begagić et al., 2023). A particularly transformative direction involves the integration of robotic systems and artificial intelligence (AI), where robotic arms could enable automated or semi-automated camera manipulation to ensure optimal visualization with minimal manual input, while AI-driven tools may provide real-time intraoperative feedback, predictive analytics, anatomical recognition, and suggested instrument trajectories, altogether enhancing precision and reducing the risk of human error. Concurrently, efforts to improve depth perception and spatial awareness through enhanced stereoscopic rendering, adjustable focal planes, and even integrated haptic feedback may significantly advance the exoscope's intraoperative functionality^29^. Augmented reality (AR) overlays capable of projecting critical anatomical landmarks, navigation data, or surgical warning zones directly into the surgical field may further support complex decision-making, especially in minimally invasive approaches (Sadagopan et al., 2024). To ensure that these innovations translate into measurable clinical benefit, large-scale prospective multicenter studies using standardized outcome metrics are necessary to evaluate the comparative effectiveness of exoscopic versus traditional microscope-assisted techniques, with particular focus on parameters such as complication rates, operative duration, postoperative recovery, and patient-reported outcomes (Ahmad and Yoon, 2022). In parallel, further research is warranted to investigate the impact of exoscope adoption on surgeon fatigue, intraoperative communication, and surgical training efficacy, areas where early experience has shown considerable promise.

The future of exoscope-assisted spinal surgery appears highly promising, with innovations in robotics, artificial intelligence, stereoscopic visualization, and augmented reality expected to optimize surgical precision, ergonomics, and intraoperative decision-making (Siller et al., 2020b; Schupper et al., 2021c). These technologies may also help reduce surgeon fatigue, improve team communication, and enhance the educational experience for trainees. At the same time, they hold the potential to expand the role of minimally invasive approaches by providing more intuitive visualization and guidance (Ahmad and Yoon, 2022; Lapevandani et al., 2025). To ensure these advances translate into meaningful clinical benefit, large prospective multicenter studies with standardized outcome measures will be essential, focusing on safety, efficiency, recovery, and patient-reported outcomes.

### 4.9 Limitations of the study

This study has some limitations. Operative time and complication rates were not systematically recorded or stratified according to different phases of the learning curve. Although surgeon-reported data suggest a short and clinically manageable adaptation period, future prospective studies with predefined outcome measures are required to objectively assess operative efficiency and complication profiles during early and late phases of exoscope adoption.

Another limitation of our study is the absence of a direct, quantitative comparison between operating microscopes and exoscopes. While we collected broad subjective feedback from the operating room team during exoscope-assisted procedures, we did not systematically collect corresponding data for microscope-assisted cases, and the microscopes available in our institution are not the latest-generation systems. Nonetheless, contemporary literature indicates that modern microscopes, including hybrid platforms, can provide visualization quality and ergonomic features that can approach those of exoscopes. Moreover, the predominance of retrospective designs and subjective assessments in the current literature limits the ability to draw definitive conclusions regarding the superiority or equivalence of exoscopes compared with microscopes.

Finally, our study did not stratify tolerance by surgeon age or correlate symptoms with operative time, which limits the ability to understand how these factors may influence surgical outcomes and adaptation to exoscope-assisted procedures.

## 5 Conclusion

This manuscript highlights the advantages of exoscope-assisted spine surgery from the perspectives of both surgeons and non-surgeons, showing clear benefits in visualization, ergonomics and freedom of movement, intraoperative communication and team engagement, and educational value through shared participation in the operating room. Consistent with the evolving literature, our observations suggest that exoscopes currently exert a greater impact on surgical workflow and training opportunities than on direct surgical outcomes, and ongoing technological refinements are likely to further expand their applicability. In the near future, the choice between an exoscope and an operating microscope may depend less on the device itself and more on surgeon preference, available resources, and the need for interdisciplinary use. Prospective comparative studies are warranted to better define clinical outcomes, cost-effectiveness, long-term benefits, and the overall impact on surgical precision, safety, and team efficiency. By sharing our firsthand experience, we hope to support informed adoption and encourage surgeons to consider this technology where it may add value in both routine and complex spinal procedures.

## Research ethics

In a report of an experiment for human subjects, it should be stated that the study was performed according to the Helsinki Declaration (http://www.wma.net/en/30publications/10policies/b3/) and approved by the Research Ethics Committee (REC) or the Institutional Review Board (IRB) of the institution where the experiment was performed. A written informed consent should be obtained from all subjects.

In cases of animal experiments, it should be stated clearly that the processes complied with regulations of institutions or national research committee related to breeding and using laboratory animals or the NIH Guide for the Care and Use of Laboratory Animals. If necessary, it can be required to submit written consents and approvals of ethics committee.

## Author contributions

Conceptualization: RR, BC.

Data curation: RR, BC, SL, PG.

Formal analysis: RR, BC, SL, PG.

Funding acquisition:

Methodology: RR, Bc.

Project administration:

Visualization: RR, BC, SL, PG.

Writing - original draft: RR, BC.

Writing - review & editing: RR, BC.

## Funding/Support

Financial support, including foundations, institutions, pharmaceutical and device manufacturers, private companies, intramural departmental sources, or any other support should be described.

## Declaration of competing interest

The authors declare that they have no known competing financial interests or personal relationships that could have appeared to influence the work reported in this paper.
