# Supplementary material for: Exoscope-assisted spine surgery: Insights from orthopedic and neurosurgical teams through a survey
Source: Brain Spine. 2026 Feb 13;6:105974. doi: 10.1016/j.bas.2026.105974 (PMC12952780; doi:10.1016/j.bas.2026.105974)
Supplement: Multimedia component 1 [file mmc1.docx]

Valutazione dell’Esoscopio in Chirurgia Vertebro-midollare

CHIRURGO

Ruolo del partecipante

Primo chirurgo - Neurochirurgo

Aiuto chirurgo – Neurochirurgo

Specializzando Neurochirurgia

Primo Chirurgo - Ortopedico

Aiuto chirurgo – Ortopedico

Specializzando Ortopedia

**A. Qualità dell’immagine e visualizzazione**

1. Come valuta la risoluzione dell’immagine fornita dall’esoscopio durante le procedure spinali?

Eccellente - Ottima - Buona - Sufficiente - Scarsa

2. Come giudica la profondità di campo e la visualizzazione delle strutture anatomiche profonde?

Eccellente - Ottima - Buona - Sufficiente - Scarsa

3. Come giudica la possibilità di ingrandimento attraverso le opzioni dello zoom offerta dall’esoscopio?

Eccellente - Ottima - Buona - Sufficiente - Scarsa

4. Come giudica la qualità dell’illuminazione del campo chirurgico?

Eccellente - Ottima - Buona - Sufficiente - Scarsa

**B. Ergonomia e affaticamento**

5. Rispetto ai tradizionali sistemi di visualizzazione (microscopio, occhiali ingranditori, etc),  l’uso dell’esoscopio ha influenzato il suo comfort fisico durante l’intervento?

Molto migliorato – Lievemente migliorato – Nessuna variazione – Lievemente peggiorato – Notevolmente peggiorato

6. Come ha tollerato l'utilizzo degli occhiali per visione 3D?

Molto bene – Bene – Neutro – Male – Molto male

7. Rispetto ai tradizionali sistemi di visualizzazione (microscopio, occhiali ingranditori, etc) l'uso dell'esoscopio ha influenzato l'affaticamento fisico durante interventi prolungati?

Molto migliorato – Lievemente migliorato – Nessuna variazione – Lievemente peggiorato – Notevolmente peggiorato

8. Quante volte ha convertito la visione dell'esoscopio con il microscopio?

Mai – Talvolta – Occasionalmente – Spesso – Sempre

**C. Comunicazione di equipe e organizzazione in sala operatoria**

9. Rispetto ai sistemi di visualizzazione tradizionali (microscopio, occhiali ingranditori, etc) in che misura la visione condivisa offerta dall’esoscopio modifica la comunicazione intraoperatoria tra i membri dell’equipe?

Molto migliorato – Lievemente migliorato – Nessuna variazione – Lievemente peggiorato – Notevolmente peggiorato

10. Rispetto ai sistemi di visualizzazione tradizionali (microscopio, occhiali ingranditori, etc) in che misura l'utilizzo dell'esoscopio ha modificato la consapevolezza di ciò che sta eseguendo il chirurgo?

Molto migliorato – Lievemente migliorato – Nessuna variazione – Lievemente peggiorato – Notevolmente peggiorato

**D. Curva di apprendimento e adozione**

11. Come descriverebbe il livello di difficoltà nell’apprendere l’utilizzo efficace dell’esoscopio?

Molto facile - Facile – Neutro – Difficile – Molto difficile

12. Come valuta la facilità d'utilizzo del braccio robotico?

Molto facile - Facile – Neutro – Difficile – Molto difficile

13. Come valuta la facilità d'utilizzo dei comandi mano/piede?

Molto facile - Facile – Neutro – Difficile – Molto difficile

14. Come valuta la funzione di sovrapposizione di immagine, in particolare in caso di tumori e/o sanguinamenti?

Molto utile – Utile – Neutro – Poco utile – Inutile

15.Come valuta la funzione di riposizionamento automatico, ad esempio dopo fluoroscopia?

Molto utile – Utile – Neutro – Poco utile – Inutile

16. Come valuta la capacità dell'esoscopio di effettuare e memorizzare video e foto?

Molto utile – Utile – Neutro – Poco utile – Inutile

17. Dopo quante procedure ha iniziato a sentirsi a proprio agio e competente nell’uso dell’esoscopio?

1-3 interventi – 4-6 interventi – 7-10 interventi – Piu’ di 10 interventi

**E. Valore formativo e didattico**

18. Rispetto ai sistemi di visualizzazione tradizionali (microscopio, occhiali ingranditori, etc) ritiene che l’esoscopio favorisca una migliore comprensione dell’anatomia spinale e delle tecniche chirurgiche durante l’intervento?

Completamente d’accordo – D’accordo – Neutro – In disaccordo – Completamente in disaccordo

19. Quanto considera efficace l’esoscopio come strumento didattico per la formazione degli specializzandi?

Molto efficace – Efficace – Neutro – Poco efficace – Per nulla efficace

20. La invitiamo a condividere eventuali commenti, suggerimenti o considerazioni aggiuntive in merito all’esperienza con l’esoscopio nella chirurgia spinale:

Testo risposta lunga
