# Supplementary material for: Exoscope-assisted spine surgery: Insights from orthopedic and neurosurgical teams through a survey
Source: Brain Spine. 2026 Feb 13;6:105974. doi: 10.1016/j.bas.2026.105974 (PMC12952780; doi:10.1016/j.bas.2026.105974)
Supplement: Multimedia component 2 [file mmc2.docx]

Valutazione dell’Esoscopio in Chirurgia Vertebro-midollare

NON CHIRURGO

Anestesista

Strumentista

Infermiere di sala

Neurofisiologo

Tecnico di radiologia

Tecnico di neurofisiopatologia

Studente

**A. Qualità dell’immagine e visualizzazione**

1. Rispetto ai tradizionali sistemi di visualizzazione (microscopio, occhiali ingranditori, ecc.) come valuta la risoluzione dell’immagine fornita dall’esoscopio durante l’intervento?

Eccellente - Ottima - Buona - Sufficiente - Scarsa

**B. Ergonomia e affaticamento**

2. Come ha tollerato l'utilizzo degli occhiali per visione 3D?

Molto bene – Bene – Neutro – Male – Molto male

**C. Comunicazione di equipe e organizzazione in sala operatoria**

3. Rispetto ai sistemi di visualizzazione tradizionali (microscopio, occhiali ingranditori, etc) in che misura la visione condivisa offerta dall’esoscopio modifica la comunicazione intraoperatoria tra i membri dell’equipe?

Molto migliorato – Lievemente migliorato – Nessuna variazione – Lievemente peggiorato – Notevolmente peggiorato

4. Rispetto ai sistemi di visualizzazione tradizionali (microscopio, occhiali ingranditori, etc) in che misura l'utilizzo dell'esoscopio ha modificato la consapevolezza di ciò che sta eseguendo il chirurgo?

Molto migliorato – Lievemente migliorato – Nessuna variazione – Lievemente peggiorato – Notevolmente peggiorato

**D. Valore formativo e didattico**

5. Rispetto ai sistemi di visualizzazione tradizionali (microscopio, occhiali ingranditori, etc) ritiene che l’esoscopio favorisca una migliore comprensione dell’anatomia spinale e delle tecniche chirurgiche durante l’intervento?

Completamente d’accordo – D’accordo – Neutro – In disaccordo – Completamente in disaccordo

6. Quanto considera efficace l’esoscopio come strumento didattico per la formazione?

Molto efficace – Efficace – Neutro – Poco efficace – Per nulla efficace

7. Rispetto ai tradizionali sistemi di visualizzazione (microscopio, occhiali ingranditori, etc), l'uso dell'esoscopio come ha modificato la sua partecipazione attiva durante la procedura chirurgica?

Molto efficace – Efficace – Neutro – Poco efficace – Per nulla efficace

8. La invitiamo a condividere eventuali commenti, suggerimenti o considerazioni aggiuntive in merito all’esperienza con l’esoscopio nella chirurgia spinale:

Testo risposta lunga
