# Supplementary material for: Exoscope-assisted spine surgery: Insights from orthopedic and neurosurgical teams through a survey
Source: Brain Spine. 2026 Feb 13;6:105974. doi: 10.1016/j.bas.2026.105974 (PMC12952780; doi:10.1016/j.bas.2026.105974)
Supplement: Multimedia component 3 [file mmc3.docx]

Evaluation of the Exoscope in Spine and Spinal Cord Surgery

NON-SURGEON

**Participant Role**

- Anesthesiologist
- Scrub Nurse
- Circulating Nurse
- Neurophysiologist
- Radiology Technician
- Neurophysiology Technician
- Student

**A. Image Quality and Visualization**

1. Compared to traditional visualization systems (microscope, loupes, etc.), how do you rate the image resolution provided by the exoscope during surgery?
   Excellent – Very good – Good – Fair – Poor

**B. Ergonomics and Fatigue**

1. How well did you tolerate the use of 3D glasses?
   Very well – Well – Neutral – Poorly – Very poorly

**C. Team Communication and Operating Room Organization**

1. Compared to traditional visualization systems (microscope, loupes, etc.), to what extent has the shared view provided by the exoscope changed intraoperative communication among team members?
   Markedly improved – Slightly improved – No change – Slightly worsened – Significantly worsened
2. Compared to traditional visualization systems (microscope, loupes, etc.), to what extent has the use of the exoscope improved your awareness of what the surgeon is performing?
   Markedly improved – Slightly improved – No change – Slightly worsened – Significantly worsened

**D. Educational and Training Value**

1. Compared to traditional visualization systems (microscope, loupes, etc.), do you think the exoscope allows for a better understanding of spinal anatomy and surgical techniques during the procedure?
   Strongly agree – Agree – Neutral – Disagree – Strongly disagree
2. How effective do you consider the exoscope as a teaching tool for training purposes?
   Very effective – Effective – Neutral – Slightly effective – Not effective at all
3. Compared to traditional visualization systems (microscope, loupes, etc.), how has the use of the exoscope influenced your active participation during the surgical procedure?
   Very effective – Effective – Neutral – Slightly effective – Not effective at all

**Additional Feedback**

1. Please share any comments, suggestions, or additional considerations regarding your experience with the exoscope in spinal surgery:
   *Open text response*
