# Supplementary material for: Exoscope-assisted spine surgery: Insights from orthopedic and neurosurgical teams through a survey
Source: Brain Spine. 2026 Feb 13;6:105974. doi: 10.1016/j.bas.2026.105974 (PMC12952780; doi:10.1016/j.bas.2026.105974)
Supplement: Multimedia component 4 [file mmc4.docx]

**Evaluation of the Exoscope in Spine and Spinal Cord Surgery**

**SURGEON (S)**

**Participant Role**

- Primary Surgeon – Neurosurgeon
- Assistant Surgeon – Neurosurgeon
- Neurosurgery Resident
- Primary Surgeon – Orthopedic Surgeon
- Assistant Surgeon – Orthopedic Surgeon
- Orthopedic Surgery Resident

**A. Image Quality and Visualization**

1. How do you rate the image resolution provided by the exoscope during spinal procedures?
   Excellent – Very good – Good – Fair – Poor
2. How do you assess the depth of field and visualization of deep anatomical structures?
   Excellent – Very good – Good – Fair – Poor
3. How do you rate the magnification options offered by the exoscope’s zoom functions?
   Excellent – Very good – Good – Fair – Poor
4. How do you rate the quality of illumination of the surgical field?
   Excellent – Very good – Good – Fair – Poor

**B. Ergonomics and Fatigue**

1. Compared to traditional visualization systems (microscope, loupes, etc.), how has the use of the exoscope affected your physical comfort during surgery?
   Markedly improved – Slightly improved – No change – Slightly worsened – Significantly worsened
2. How well did you tolerate the use of 3D glasses?
   Very well – Well – Neutral – Poorly – Very poorly
3. Compared to traditional visualization systems (microscope, loupes, etc.), how has the use of the exoscope affected physical fatigue during prolonged procedures?
   Markedly improved – Slightly improved – No change – Slightly worsened – Significantly worsened
4. How often did you switch from the exoscope to the microscope during a procedure?
   Never – Rarely – Occasionally – Often – Always

**C. Team Communication and Operating Room Organization**

1. Compared to traditional visualization systems (microscope, loupes, etc.), to what extent has the shared view offered by the exoscope modified intraoperative communication among team members?
   Markedly improved – Slightly improved – No change – Slightly worsened – Significantly worsened
2. Compared to traditional visualization systems (microscope, loupes, etc.), to what extent has the use of the exoscope modified the awareness of what the surgeon is performing?
   Markedly improved – Slightly improved – No change – Slightly worsened – Significantly worsened

**D. Learning Curve and Adoption**

1. How would you describe the level of difficulty in learning to use the exoscope effectively?
   Very easy – Easy – Neutral – Difficult – Very difficult
2. How do you assess the ease of use of the robotic arm?
   Very easy – Easy – Neutral – Difficult – Very difficult
3. How do you assess the ease of use of the hand/foot controls?
   Very easy – Easy – Neutral – Difficult – Very difficult
4. How do you rate the image overlay function, particularly in cases of tumors and/or bleeding?
   Very useful – Useful – Neutral – Slightly useful – Not useful
5. How do you rate the automatic repositioning function (e.g., after fluoroscopy)?
   Very useful – Useful – Neutral – Slightly useful – Not useful
6. How do you rate the exoscope’s ability to record and store videos and photos?
   Very useful – Useful – Neutral – Slightly useful – Not useful
7. After how many procedures did you begin to feel comfortable and competent in using the exoscope?
   1–3 procedures – 4–6 procedures – 7–10 procedures – More than 10 procedures

**E. Educational and Training Value**

1. Compared to traditional visualization systems (microscope, loupes, etc.), do you think the exoscope promotes a better understanding of spinal anatomy and surgical techniques during the procedure?
   Strongly agree – Agree – Neutral – Disagree – Strongly disagree
2. How effective do you consider the exoscope as a teaching tool for resident training?
   Very effective – Effective – Neutral – Slightly effective – Not effective at all

**Additional Feedback**

1. Please share any comments, suggestions, or additional considerations regarding your experience with the exoscope in spinal surgery:
   *Open text response*
